# Assessment of Palliative Care Needs in Non-oncological Patients in Primary Health Care: A Cross-Sectional Study

**DOI:** 10.7759/cureus.91809

**Published:** 2025-09-07

**Authors:** Maria José Vilas Boas Machado, Ana Rita Oliveira, Gabriela Guiomar, Catarina Roteia, Carolina Balao, Nadine Santos, Anabela Silva, Jorge Hêrnani-Eusébio

**Affiliations:** 1 School of Medicine, Universidade do Minho, Braga, PRT; 2 Unidade de Saúde Familiar Sanus Carandá, Unidade Local de Saúde de Braga, Braga, PRT; 3 Unidade de Saúde Familiar Braga Norte, Unidade Local de Saúde de Braga, Braga, PRT; 4 Unidade de Saúde Familiar Saúde Oeste, Unidade Local de Saúde de Braga, Braga, PRT; 5 Unidade de Saúde Familiar Gualtar, Unidade Local de Saúde de Braga, Braga, PRT; 6 Life and Health Sciences Research Institute, Universidade do Minho, Braga, PRT; 7 Unidade de Saúde Familiar Sá de Miranda, Unidade Local de Saúde de Braga, Braga, PRT

**Keywords:** advanced care planning, chronic disease, clinical decision-making, needs assessment, palliative care

## Abstract

Introduction

Patients with non-oncological chronic illnesses often have significant palliative care needs (PCN), which frequently go unrecognized due to the unpredictable course of these diseases. Early identification tools can facilitate the detection of PCN within primary health care (PHC). This study aimed to assess PCN among patients with stroke, chronic obstructive pulmonary disease, heart failure, and dementia in four primary care units (PCUs) of the Braga Local Health Unit (ULS Braga); to compare the Surprise Question (SQ) with the Portuguese version of the Supportive and Palliative Care Indicators Tool (SPICT-PT™); to evaluate the level of agreement between family physicians (FPs) and family nurses (FNs); and to characterize referrals to palliative care (PC) units.

Materials and methods

A cross-sectional observational study was conducted, including 327 chronic patients from four PCUs of ULS Braga. Both FPs and FNs applied the SQ and the SPICT-PT™ to assess each patient’s PCN.

Results

PCN were identified in 44.0% of patients using the SQ and in 38.2% using the SPICT-PT™, with a statistically significant correlation between the tools (*p *< 0.001). Dementia showed the strongest association with PCN. There was moderate agreement between FPs and FNs for both tools, with FPs reporting greater confidence in using the SPICT-PT™. Despite the identification of needs, only 12.5% (SQ) and 14.4% (SPICT-PT™) of patients were referred to PC units.

Conclusions

Non-oncological chronic patients, particularly those with dementia, often have unmet PCN. Routine use of structured tools can enhance early identification, support multidisciplinary evaluation, and highlight the need for better integration of PC into PHC.

## Introduction

According to the World Health Organization, palliative care (PC) aims to improve the quality of life of individuals with life-threatening illnesses and their families through the early identification and appropriate treatment of pain and other physical, psychological, or spiritual problems [1]. Yearly, approximately 56.8 million people worldwide are estimated to have PC needs (PCN), yet only 14.0% receive adequate support [1]. In Portugal, despite this right being enshrined in legislation, only 30.0% of the 100,000 patients in need have access to it [2,3].

Patients with non-oncological chronic diseases are particularly affected. Although their needs are similar to those of cancer patients, their access to PC is often more delayed and limited. This scenario is exacerbated by increased longevity and the growing prevalence of chronic diseases, accounting for 75.0% of deaths in developed countries [4,5]. The lack of early recognition of PCN in these patients, often due to unpredictable disease progression, leads to delays in palliative intervention [6]. It is estimated that 69-82% of patients with advanced chronic illnesses will require PC [7].

Respiratory, cardiovascular, cerebrovascular, and neurodegenerative diseases are among the main causes of PCN worldwide [1]. In Portugal, cerebrovascular diseases are the leading cause of mortality and disability, followed by cardiovascular diseases, chronic obstructive pulmonary disease (COPD), and dementia. The National Health Plan 2021-2030 acknowledges the need to reduce their burden of mortality and disability [8].

PC has evolved from focusing solely on terminal cancer patients to the care of individuals with various chronic and life-limiting conditions [1]. The fluctuating trajectory of chronic diseases requires an integrated approach that combines curative treatments and PC, making the early identification of PCN challenging [9]. Therefore, it is essential to provide healthcare professionals with tools to systematically identify these patients.

The Surprise Question (SQ), “Would you be surprised if this patient were to die in the next year?”, is a tool used to identify patients who may benefit from PC. A negative response should trigger the adoption of a palliative approach [10]. The SQ has been translated and validated for the Portuguese population, with a sensitivity of 63.9% and a specificity of 67.3% when used by family physicians (FPs) in patients with advanced chronic diseases [11]. It has limitations related to the subjectivity of professional judgment, but it is notable for its simplicity and ease of integration into clinical practice.

In this context, the Supportive and Palliative Care Indicators Tool (SPICT™), created in 2010 in Scotland and translated into Portuguese, SPICT-PT™, emerges as another useful tool for identifying patients with PCN while also providing guidance for care planning [12]. With a sensitivity of 75.5% and a specificity of 68.6%, based on data currently only available in an unpublished national master’s thesis, SPICT-PT™ stands out for its intuitive application and clear language, allowing healthcare professionals to use it efficiently during consultations (Unpublished master’s thesis: Mira M. Translation and Validation of the SPICT™ (Supportive & Palliative Care Indicators Tool) Scale for the Portuguese Population [Article in Portuguese]. Universidade de Lisboa; 2020) [13].

The provision of PC is considered an integral part of the competencies of primary health care (PHC). Consequently, it is essential that healthcare professionals recognize their central role in identifying and delivering palliative actions and referring patients to PC teams [14]. The systematic use of tools such as the SQ and SPICT-PT™ may facilitate the early identification of PCN within PHC, improving community access.

Despite the relevance of the SQ and SPICT-PT™ tools in identifying PCN, studies assessing their systematic application in PHC settings in Portugal remain scarce [15,16]. Additionally, evidence regarding agreement between different professional groups, particularly FPs and family nurses (FNs), in the use of these tools is limited [17]. This gap in the literature hinders the definition of effective strategies for implementing and evaluating interventions aimed at the early detection of PCN.

The main objective of this study was to determine the prevalence of PCN in patients with stroke, COPD, heart failure (HF), or dementia in PHC.

This article was previously presented as an oral communication at the 42nd National Meeting of General and Family Medicine on March 29, 2025.

## Materials and methods

Study design and setting

Between September 15 and 30, 2024, an observational, cross-sectional study was carried out in four urban and rural primary care units within the Braga Local Health Unit (ULS Braga). A nonrandomized convenience sampling method was used based on inclusion and exclusion criteria.

Population

A nonrandomized convenience sampling method was applied, with eligibility determined according to predefined criteria. Inclusion criteria comprised patients with a recorded diagnosis of stroke, COPD, HF, or dementia and at least one in-person consultation with their FPs and/or FNs in the six months prior to data collection. Exclusion criteria included patients under 18 years; active oncological disease or cancer diagnosis within the past five years; and diagnosis of severe psychiatric disorders (bipolar disorder, schizophrenia, or psychotic disorders). Eligible patients were identified by FPs using the Functional Units Information and Monitoring Module (MIM@UF®) and the following International Classification of Primary Care, second edition (ICPC-2) codes: K90 (Thrombosis/Stroke), K91 (Cerebrovascular Disease), R95 (COPD), K77 (HF), and P70 (Dementia).

Study objectives

The primary objective of this study was to determine the prevalence of PCN in patients with stroke, COPD, HF, or dementia in PHC. The secondary objectives were to assess the agreement between FPs and FNs in PCN identification; to compare the SQ and SPICT-PT™ in detecting PCN; to determine and compare the prevalence of PCN for each selected disease; and to evaluate the referral of patients with PCN to PC teams.

Variables and data sources

Data were extracted from the SClínico® electronic medical records using a structured form completed independently by both the FP and the FN. The form comprised three sections: the SQ, “Would you be surprised if this patient were to die in the next year?”, with respondents also rating their confidence on a 10-point Likert scale (1 = not at all confident; 10 = completely confident); the SPICT-PT™ tool, which includes general and disease-specific indicators of health decline, with a positive result defined as the presence of at least two general and one clinical indicator, suggesting PCN, and for which confidence in its use was also recorded; and referral information, comprising data obtained from the National Health Service and the National Integrated Continued Care Network, including the type of team involved and their response.

No training sessions or formal standardization procedures were conducted before the study; professionals completed the tools according to their clinical practice and judgment.

Bias control

Potential bias was minimized through the independent completion of the SQ and SPICT-PT™ by both the FP and the FN, as well as the use of a standardized data extraction form to ensure consistency in data collection.

Statistical analysis

Data analysis was performed using IBM SPSS Statistics for Windows, Version 29.0 (Released 2022; IBM Corp., Armonk, NY, USA). A descriptive analysis of the study variables was conducted, calculating absolute (n) and relative (%) frequencies for qualitative variables and mean, median, SD, and IQR for quantitative variables. The IQR was reported as the width of the distribution, calculated as the difference between the 75th and 25th percentiles (Q3-Q1). Normality of the quantitative variables was assessed through analysis of kurtosis and skewness values, visual inspection of histograms, and the Kolmogorov-Smirnov normality test.

The chi-square test (χ²) was used to analyze the association between the SQ response and the SPICT-PT™ result for each patient, as well as the association between diagnoses and the results of these tools. With less than 20% of cells having expected frequencies below five, Fisher’s exact test was not applied. Phi (Φ) was used as a measure of effect size. Absolute values of 0.10, 0.30, and 0.50 were considered to represent small, moderate, and large effect sizes, respectively.

Cohen’s kappa test (κ) was used to determine the level of agreement between the FPs and FNs responses using both tools, classified as follows: ≤0: no agreement; 0.01-0.20: slight or no agreement; 0.21-0.40: fair agreement; 0.41-0.60: moderate agreement; 0.61-0.80: substantial agreement; and 0.81-1.00: almost perfect agreement.

Significant differences in perceived confidence when using the tools to identify PCN were determined using the nonparametric Wilcoxon Signed Rank test for variables that did not meet normality criteria. Subsequently, the parametric paired Student’s t-test was applied. When the results of both tests were concordant, the parametric test was considered valid, and the mean and SD of each group were reported. Cohen’s d was used to estimate the effect size in the Student’s t-test. Effect sizes were classified as small (0.20), moderate (0.50), and large (0.80).

For all analyses, a p-value < 0.05 was considered statistically significant, and a 95% confidence interval was used.

Ethical approval

This study received approval from the Ethics Committee for Research in Life and Health Sciences and the Health Ethics Committee of the ULS Braga.

## Results

Demographics and prevalence of conditions

During the established period, 21 FPs and 19 FNs participated in this study, yielding a sample of 327 patients after applying inclusion and exclusion criteria. Of these, 171 (52.3%) were female and 156 (47.7%) were male, with a median age of 78 years (IQR 17; range 43-99).

The most prevalent condition was COPD, affecting 119 (36.4%) patients, followed by HF (92; 28.1%) and stroke (88; 26.9%). Dementia was the least prevalent, diagnosed in 66 (20.2%) patients. It is noteworthy that 36 (11.0%) patients had two concurrent diagnoses, and one (0.1%) had three.

SPICT-PT™ and SQ results

Descriptive variables of the SPICT-PT™ are presented in Table 1. Among the general indicators of poor or deteriorating health, the most frequently identified were dependence on personal care and/or increased need for caregiver support, noted in 90 (27.5%) patients by FPs and 97 (29.7%) by FNs. Both professional groups identified only one (0.3%) patient who had requested PC or preferred to prioritize quality of life over curative treatments.

**Table 1:** Descriptive variables and SPICT-PT™ results n = absolute frequency Values in bold represent the most frequent clinical indicator of life-shortening conditions for each diagnostic group. FN, family nurse; FP, family physician; GFR, glomerular filtration rate; HF, heart failure; PC, palliative care; SPICT-PT™, Supportive and Palliative Care Indicators Tool, Portuguese version

	FP		FN	
	No, n (%)	Yes, n (%)	No, n (%)	Yes, n (%)
General indicators of poor or deteriorating health				
Urgent or emergency hospital admission(s) or visits	264 (80.7)	63 (19.3)	254 (77.7)	73 (22.3)
Functional ability is poor or deteriorating, with limited reversibility	281 (85.9)	46 (14.1)	260 (79.5)	67 (20.5)
Depends on others more for care due to increasing physical and/or mental health problems; the person’s carer needs more help and support	237 (72.5)	90 (27.5)	230 (70.3)	97 (29.7)
Progressive weight loss; remains underweight; low muscle mass	307 (93.9)	20 (6.1)	281 (85.9)	46 (14.1)
Persistent symptoms despite optimal treatment of health condition(s)	266 (81.3)	61 (18.7)	253 (77.4)	74 (22.6)
The person (or family) asks for PC; chooses to reduce, stop, or not have treatment; or wishes to focus on quality of life	326 (99.7)	1 (0.3)	326 (99.7)	1 (0.3)
Clinical indicators of life-shortening conditions				
Cancer				
Functional ability deteriorating due to progressive cancer	0 (0.0)	0 (0.0)	0 (0.0)	0 (0.0)
Too frail for cancer treatment, or treatment is for symptoms	0 (0.0)	0 (0.0)	0 (0.0)	0 (0.0)
Dementia or frailty				
Unable to dress, walk, or eat without help	272 (83.2)	55 (16.8)	255 (78.0)	72 (22.0)
Eating and drinking less; difficulty with swallowing	307 (93.9)	20 (6.1)	299 (91.4)	28 (8.6)
Urinary and fecal incontinence	298 (91.1)	29 (8.9)	275 (84.1)	52 (15.9)
Not able to communicate by speaking; little social interaction	303 (92.7)	24 (7.3)	288 (88.1)	39 (11.9)
Frequent falls; fractured femur	318 (97.2)	9 (2.8)	307 (93.9)	20 (6.1)
Recurrent febrile illnesses or infections; aspiration pneumonia	320 (97.9)	7 (2.1)	311 (95.1)	16 (4.9)
Neurological disease				
Progressive deterioration in physical and/or cognitive function despite optimal therapy	265 (81.0)	62 (19.0)	252 (77.1)	75 (22.9)
Speech problems with increasing difficulty communicating and/or progressive difficulty with swallowing	320 (97.9)	7 (2.1)	312 (95.4)	15 (4.6)
Recurrent aspiration pneumonia; breathless or respiratory failure	323 (98.8)	4 (1.2)	309 (94.5)	18 (5.5)
Ongoing disability with worsening physical and/or mental health after a major stroke or multiple strokes	320 (97.9)	7 (2.1)	306 (93.6)	21 (6.4)
Heart or vascular disease				
HF or extensive, untreatable coronary artery disease; breathlessness or chest pain at rest or on minimal effort	305 (93.3)	22 (6.7)	292 (89.3)	35 (10.7)
Severe, inoperable peripheral vascular disease	319 (97.6)	8 (2.4)	321 (98.2)	6 (1.8)
Respiratory disease				
Severe, long-term lung disease; breathlessness at rest or on minimal effort between exacerbations	305 (93.3)	22 (6.7)	305 (93.3)	22 (6.7)
Persistent hypoxia needing long-term oxygen therapy	324 (99.1)	3 (0.9)	322 (98.5)	5 (1.5)
Has needed ventilation for respiratory failure, or ventilation is contraindicated	322 (98.5)	5 (1.5)	313 (95.7)	14 (4.3)
Kidney disease				
Stage 4 or 5 chronic kidney disease (eGFR < 30ml/min) with deteriorating health	322 (98.5)	5 (1.5)	321 (98.2)	6 (1.8)
Kidney failure complicating other life-shortening conditions or treatments	324 (99.1)	3 (0.9)	325 (99.4)	2 (0.6)
Stopping or not starting dialysis	0 (0.0)	0 (0.0)	0 (0.0)	0 (0.0)
Liver disease				
Cirrhosis with one or more complications in the past year	0 (0.0)	0 (0,0)	326 (99.7)	1 (0.3)
Liver transplant is not possible	0 (0.0)	0 (0.0)	0 (0.0)	0 (0.0)
Other conditions				
Deteriorating with physical or mental illnesses, multiple conditions, and/or complications that are not reversible	323 (98.8)	4 (1.2)	320 (97.9)	7 (2.1)
Positive SPICT™ result	248 (75.8)	79 (24.2)	218 (66.7)	109 (33.3)

Regarding clinical indicators of life-shortening conditions, progressive deterioration in physical and/or cognitive function was the most common criterion, applicable to 62 (19.0%) patients by FPs and to 75 (22.9%) by FNs. No cancer-related criteria were considered. The criteria “stopping or not starting kidney dialysis” and “liver transplant is not possible” were also not selected. FPs assigned a positive SPICT-PT™ result to 79 (24.2%) patients, while FNs did so for 109 (33.3%). In total, 125 (38.2%) patients had a positive SPICT-PT™ result in at least one of the assessments. Regarding the SQ, a total of 144 (44.0%) patients would not surprise at least one of the professionals if they died within a year (FPs: 33.3%; FNs: 34.9%).

Table 2 presents the correspondence between responses to the SQ and SPICT-PT™ results for each patient. Among cases where FPs answered “No” to the SQ, 61.5% had a positive SPICT-PT™, indicating agreement on the presence of PCN. Agreement on the absence of PCN occurred in 94.5% of cases. The chi-square test revealed a statistically significant association between the FP’s SQ response and the SPICT-PT™ result (p < 0.001, Φ = -0.616), indicating a strong effect size.

**Table 2:** Relationship between the SQ response and the SPICT-PT™ results n = absolute frequency; χ² = chi-square test; Φ = phi coefficient FN, family nurse; FP, family physician; SPICT-PT™, Supportive and Palliative Care Indicators Tool, Portuguese version; SQ, Surprise Question

	Would you be surprised if this patient were to die in the next year?		Chi-square test value	p-value	Effect size
	No, n (%)	Yes, n (%)			
FP					
SPICT-PT™negative	42 (38.5)	206 (94.6)	χ² = 124.211	<0.001	Φ = -0.616
SPICT-PT™positive	67 (61.5)	12 (5.5)			
FN					
SPICT-PT™ negative	36 (31.6)	182 (85.4)	χ²= 96.961	<0.001	Φ = -0.545
SPICT-PT™ positive	78 (68.4)	31 (14.6)			

For FNs, 68.4% of patients with a “No” response to the SQ had a positive SPICT-PT™ result. Likewise, 85.4% of patients whom FNs would be surprised to see die had a negative SPICT-PT™ result. The association between FNs’ SQ responses and SPICT-PT™ results was also statistically significant and strong (p < 0.001, Φ = -0.545).

The comparison between professional groups revealed moderate agreement in SQ responses (κ = 0.558; p < 0.001) and SPICT-PT™ results (κ = 0.542; p < 0.001) (Table 3).

**Table 3:** Concordance between SQ and SPICT-PT™ results by FPs and FNs k = Cohen’s kappa FN, family nurse; FP, family physician; SPICT-PT™, Supportive and Palliative Care Indicators Tool, Portuguese version; SQ, Surprise Question

Would you be surprised if this patient were to die in the next year?					
		FN		Cohen’s kappa value	p-value
		No	Yes	k = 0.558	<0.001
FP	No	79	30		
	Yes	35	183		
SPICT-PT™Result					
		FN		Cohen’s kappa value	p-value
		Negative	Positive	k = 0.542	<0.001
FP	Negative	202	46		
	Positive	16	63		

Confidence in using PCN identification tools

FPs reported significantly higher confidence when applying the SPICT-PT™ compared to answering the SQ (p = 0.005, d = -0.156). In contrast, FNs showed no significant difference in confidence between the two tools (p = 0.279, d = -0.060), with small effect sizes in both cases (Table 4).

**Table 4:** Assessment of perceived confidence in using PCN identification tools according to professional group t = Student’s t-test for paired samples; d = Cohen’s d FN, family nurse; FP, family physician; PCN, palliative care needs; SPICT-PT™, Supportive and Palliative Care Indicators Tool, Portuguese version; SQ, Surprise Question

	Mean	SD	t-value	p-value	Effect size
FP					
SQ	7.77	1.938	t = -2.823	0.005	d = -0.156
SPICT-PT™	7.96	1.719			
FN					
SQ	8.22	1.817	t = -1.084	0.279	d = -0.060
SPICT-PT™	8.32	1.885			

PCN and referrals

According to information provided by FPs and FNs, based on patient medical records, only 18 (12.5%) of patients with PCN identified via the SQ and 14.4% of those identified via the SPICT-PT™ had been referred to a PC team. Of the referred patients, eight (44.4%) had a diagnosis of dementia, six (33.3%) of HF, three (16.6%) of stroke, and three (16.6%) of COPD.

Among referred patients, the SQ and SPICT-PT™ results were generally consistent, except in three (16.7%). A total of 15 (83.3%) patients were referred to the Community Support PC Team, two (11.1%) to PC Units (National Network for Integrated Continuous Care), and one (5.6%) to the In-Hospital PC Support Team. Of these, three (16.7%) were rejected, one by each team.

Regarding the correlation between patient diagnoses and potential PCN, only the presence of COPD and dementia significantly influenced the responses from both professionals to the SQ (Table 5) and the SPICT-PT™ (Table 6). A significant association was observed between the presence of COPD and the absence of PCN. Conversely, the presence of dementia was significantly associated with the presence of PCN.

**Table 5:** Prevalence of PCN identified via the SQ by chronic disease according to FPs and FNs n = absolute frequency; χ² = chi-square test; Φ = phi coefficient COPD, chronic obstructive pulmonary disease; FN, family nurse; FP, family physician; HF, heart failure; PCN, palliative care needs; SPICT-PT™, Supportive and Palliative Care Indicators Tool, Portuguese version; SQ, Surprise Question

Would you be surprised if this patient were to die in the next year?						
	Total	No	Yes	Chi-square test value	p-value	Effect size
FP						
COPD, n (%)	119 (36.4)	24 (20.2)	95 (79.8)	χ² = 14.592	<0.001	Φ = 0.211
HF, n (%)	92 (28.1)	37 (40.2)	55 (59.8)	χ² = 2.730	0.098	Φ = -0.091
Stroke, n (%)	88 (26.9)	20 (22.7)	68 (77.3)	χ² = 6.095	0.014	Φ = 0.137
Dementia, n (%)	66 (20.2)	44 (66.7)	22 (33.3)	χ² = 41.345	<0.001	Φ = -0.356
FN						
COPD, n (%)	119 (36.4)	29 (24.4)	90 (75.6)	χ² = 9.070	0.003	Φ = 0.167
HF, n (%)	92 (28.1)	34 (37.0)	58 (63.0)	χ² = 0.247	0.619	Φ = -0.027
Stroke, n (%)	88 (26.9)	26 (29.5)	62 (70.5)	χ² = 1.499	0.22	Φ = 0.068
Dementia, n (%)	66 (20.2)	41 (62.1)	25 (37.9)	χ²= 27.057	<0.001	Φ = -0.288

**Table 6:** Prevalence of PCN according to SPICT-PT™ by chronic disease n = absolute frequency; χ² = chi-square test; Φ = phi coefficient COPD, chronic obstructive pulmonary disease; HF, heart failure; PCN, palliative care needs; SPICT-PT™, Supportive and Palliative Care Indicators Tool, Portuguese version

SPICT-PT^TM^ result						
	Total	Negative	Positive	Chi-square test value	p-value	Effect size
FP						
COPD, n (%)	119 (36.4)	103 (86.6)	16 (13.4)	χ² = 11.720	<0.001	Φ = -0.189
HF, n (%)	92 (28.1)	73 (79.3)	19 (20.7)	χ² = 0.859	0.354	Φ = -0.051
Stroke, n (%)	88 (26.9)	67 (76.1)	21 (23.9)	χ² = 0.006	0.940	Φ = 0.004
Dementia, n (%)	66 (20.2)	29 (43.9)	37 (56.1)	χ²= 45.930	<0.001	Φ = 0.375
FN						
COPD, n (%)	119 (36.4)	97 (81.5)	22 (18.5)	χ²= 18.555	<0.001	Φ = -0.238
HF, n (%)	92 (28.1)	62 (67.4)	30 (32.6)	χ² = 0.030	0.862	Φ = -0.010
Stroke, n (%)	88 (26.9)	59 (67.0)	29 (33.0)	χ² = 0.008	0.903	Φ = -0.005
Dementia, n (%)	66 (20.2)	18 (27.3)	48 (72.7)	χ² = 57.746	<0.001	Φ = 0.420

## Discussion

Early PC intervention is essential for chronic patients, improving quality of life by managing symptoms and providing social, psychological, and spiritual support [1]. However, the early identification of PCN in non-oncological patients is challenging in PHC due to unpredictable disease progression [9]. Structured tools can help healthcare professionals identify and refer these patients.

This study aimed to estimate the prevalence of PCN in high-mortality chronic non-oncological diseases in Portugal, compare the SQ with the SPICT-PT™, and assess professionals’ confidence using them. COPD was the most common condition, followed by HF, stroke, and dementia. This differs from general Portuguese population data, where stroke is more prevalent, followed by COPD, HF, and dementia [18-21]. This may reflect specific care needs in ULS Braga or a focus on respiratory diseases in this region.

Regarding PCN identification, 44.0% of patients were considered unlikely to surprise their FPs or FNs if they died within a year. This is lower than rates reported in other Portuguese and inpatient studies [11,22]. The SPICT-PT™ identified PCN in 38.2% of patients, higher than reported in Japan (9.2%) and Portugal (7.2%) [23,24], likely because these studies did not exclusively focus on non-oncological chronic patients. A rural Indian study reported 16.7%, closer to our results [25].

The most frequent general indicator in SPICT-PT™ was “dependence on personal care or caregiver support,” followed by “persistent symptoms despite treatment,” consistent with previous literature (Unpublished master’s thesis: Mira M, 2020) [26]. Among clinical indicators, progressive physical or cognitive decline was most frequent, followed by difficulty with basic activities of daily living [27]. As in other studies [28], agreement between SQ and SPICT-PT™ was good, supporting their complementary use: the SQ as a quick screen and the SPICT-PT™ for detailed assessment [29].

FPs and FNs showed moderate agreement on SQ answers, consistent with previous studies in Portugal [17] and Spain [5]. Nonetheless, other studies report differences in subjective PCN assessment between professional groups. These studies reported greater accuracy among physicians compared to nurses in predicting 12-month mortality [26,30].

For SPICT-PT™, agreement between FPs and FNs was consistent with prior reliability studies [31,32]. FPs reported greater confidence in its use, possibly due to its objective structure, as also seen in other research [33]. Other studies found that nurses may need clarification of certain clinical terms in SPICT™, and some physicians find the tool complex [34]. In our study, nurses’ confidence did not differ between tools, possibly reflecting their close and continuous patient follow-up.

An important finding was the low rate of referral to specialized PC teams: only 12.5% (SQ) and 14.4% (SPICT-PT™) of identified patients were referred, similar to other reports showing five to 25% referral rates [17,23,35]. This underscores the need for better integration between PHC and PC services. Notably, three referrals were rejected by the PC team, and the lack of documented reasons for these rejections represents a limitation of the present analysis.

The diagnostic analysis revealed a significant association between COPD, a positive SQ response, and a negative SPICT-PT™ result, suggesting an absence of PCN. Contrary to findings from studies in different socioeconomic contexts, such as a rural area in Nepal [36]. In contrast, dementia showed a significant association with the presence of PCN, consistent with earlier studies and being the most frequently referred diagnosis [23,24,37].

Limitations include using a convenience sample, which limits generalizability, and the older age of participants, which may confound results given PCN’s association with age [38]. The SPICT-PT™ is still undergoing full validation in Portugal, potentially affecting reliability (Unpublished master’s thesis: Mira M, 2020) [39]. Reliance on records and recall without full clinical examination may also have introduced bias. A further limitation is that no formal training or standardization session was provided to FPs and FNs prior to data collection. Although both groups had experience in chronic disease management and familiarity with the SQ, and the SPICT-PT™ was applied according to its Portuguese guidance, the lack of structured preparation may have introduced some variability.

Despite these limitations, this study provides valuable data on the reality of PHC in Portugal, emphasizing the importance of systematic screening and better coordination with specialized PC teams. Future research should involve larger, more diverse samples to strengthen findings.

## Conclusions

This study confirmed a high prevalence of PCN among non-oncological chronic patients in primary care, particularly in those with dementia. It highlights the value of using tools like the SQ and SPICT-PT™ to support early and systematic identification of PCN. The moderate agreement between FPs and FNs reinforces the importance of multidisciplinary assessment and the complementarity of these tools. Despite identifying many patients with PCN, referral rates to PC teams were low, revealing a gap between the identification of needs and the actual care response. These findings underscore the need to strengthen coordination between PHC and specialized PC teams, promote continuous training for healthcare professionals, and implement standardized assessment and referral protocols.

Integrating PC into PHC using reliable tools can support more patient-centered, timely, and equitable care planning, better addressing the complex needs of patients with chronic non-oncological conditions.
